# Supplementary material for: Are carbon emissions trading and green financial instruments synergistic? -Comprehensive quantitative research based on content analysis
Source: PLoS One. 2024 Mar 7;19(3):e0298601. doi: 10.1371/journal.pone.0298601 (PMC10919601; doi:10.1371/journal.pone.0298601)
Supplement: S1 Data — (ZIP) [file pone.0298601.s001.zip › Data/地级市Z_F工作报告.zip!/╣π╢1⁄2/╟σ╘╢╩╨/└┤╫╘╣1⁄2╓┌║┼í╛┬φ┐╦╩2╛▌═°í┐_2018.docx]

各位代表：

我代表市人民政府，向大会作工作报告，请予审议，并请政协委员和其他列席人员提出意见。

2017年工作回顾

过去一年，是全市上下践行新发展理念，深化转型攻坚，实现本届政府良好开局的重要一年。我们在省委、省政府和市委的正确领导下，全面贯彻习近平新时代中国特色社会主义思想，紧紧围绕“南融北康”发展目标，大力实施广清一体化战略，深化供给侧结构性改革，突出抓好重点项目重大平台建设，经济社会发展取得新成效、迈出新步伐，基本完成市七届人大一次会议确定的目标任务。

一年来，我们着力稳增长促转型，突出抓好创新驱动，经济发展稳中提质。

经济保持平稳增长。预计全市完成生产总值1500亿元，比上年增长（以下简称增长）6%；完成固定资产投资670亿元，增长8%；规上工业增加值438亿元，增长3.5%；农林牧渔业增加值223亿元，增长4.9%；一般公共预算收入103亿元，可比口径增长11%，非税比重下降5.8个百分点；社会消费品零售总额683亿元，增长9%；外贸进出口325亿元，增长11.5%，其中出口增长5.2%，实际利用外资增长34.3%；金融机构本外币存款、贷款余额分别增长12.8%和19.5%。

转型升级步伐加快。现代服务业、高技术制造业和先进制造业增加值分别增长10%、10%和7%。单位GDP电耗、能耗分别下降13%、5.5%。完成工业技改投资62亿元，增长27%；16家企业开展“机器换人”。新增规上企业16家。规上工业实现利润增长13%。修订完善扶持企业上市政策，5家企业启动IPO，7家企业在“新三板”挂牌，90多家企业在区域性股权交易中心挂牌。大力发展旅游和商贸服务业，获评“中国最具特色旅游城市”，全市列入国家、省全域旅游示范区创建单位8个，新增4A级景区1个，旅游接待人次和收入分别增长14%、16%；世纪荟、时代广场等商业综合体建成营业；广百南部物流园正式动工。

创新发展取得实效。制定“创新十条”，创新发展财政投入力度居粤东西北前列，累计发放科技创新券2594万元。清远国家高新区在全国排名上升2位，成为国家高性能金属材料高新技术产业化基地。新增高新技术企业64家、省级以上企业工程中心42家，3家新材料产业技术联盟获省认定，实现主营业务收入5亿元以上工业企业研发机构全覆盖。拥有省“扬帆计划”创新创业团队6个，数量居粤东西北第一。天安智谷、华大电商、金创等“双创”平台建设进展顺利。成功举办首届清远职业技能大赛。我市企业参与制订国家标准15项。专利授权量、发明专利授权量分别增长16%和20%。天安绿色创投基金和首个“碳金融”项目落地。建成电商产业园8个。华南声谷动工建设。

一年来，我们着力区域协调发展，突出抓好产业共建，“南融北康”迈上新水平。

产业共建深入推进。广清新一轮帮扶有序开展。成功举办面向广州、深圳和省属国有企业三场招商推介会，签约项目56个，计划投资1170.6亿元。全市新引进工业项目178个；承接珠三角梯度转移项目56个，完成省定任务的112%，其中产业共建亿元以上项目35个。园区平台加快发展，广清产业园清城片区“三纵三横”路网基本建成，办公、排污等配套设施投入使用，新签约项目51个，累计落地动工项目62个，试（投）产项目26个；佛冈片区加快推进“三通一平”；清新片区启动前期工作。全市园区完成规上工业增加值141.8亿元、税收27.2亿元，分别增长19.6%和28.5%。广清一体化首批3条跨市公交线路正式开通。积极参与广佛肇清云韶经济圈建设。

北部发展条件改善。积极推进粤北生态特别保护区建设前期工作。广连高速、二广与清连高速连接线动工建设，连州通用机场完成场址初选，国道323线连山、连南县城段改建工程加快，德建水库完成工程量的75.5%，连山人民医院完成整体搬迁，连州摄影博物馆落成开馆。绿色能源和生态农业、旅游等加快发展，连南生态农业光伏发电一期20兆瓦项目并网发电，连州福山风电和阳山桔子塘风电、生物质发电等项目稳步推进，阳山创建为全省首个“国家生态原产地产品保护示范区”、获评“全国森林旅游示范县”，连州连续三年成为全省县（市）域旅游十强，连山戏水节获评全国“2017年最具影响力特色节庆”，连南稻田鱼文化节被认定为“国家级示范性渔业文化节庆”。民族工业园合作共建机制逐步完善，规上工业增加值、税收分别增长25%和30%。

重大项目扎实推进。重点项目完成投资376.6亿元，完成年度计划投资的89.5%。清远长隆项目建设扎实推进，完成投资15亿元；清晖南路延伸段、森林大道等配套道路加快建设。省级职教基地建设取得新突破，五所院校全部完成立项，广东建设、交通、科贸三所院校动工建设。“高接高”、汕昆、汕湛高速清远段全面开工，全市交通基础设施建设完成投资110亿元，投资额为历年最高。国内首条中低速磁浮旅游专线动工建设。广清城轨一期线下工程基本完工。北江航道扩能升级工程完成年度任务。积极推进广清永高铁前期规划。英德通用机场纳入省“十三五”建设项目。潖江蓄滞洪区项目完成可研报告并列入省2018年重点推进项目。

一年来，我们着力优化发展环境，突出抓好综合改革，发展活力不断增强。

供给侧改革取得实效。加快推进“去降补”，淘汰钢铁落后产能500万吨；商品房销售面积941.1万平方米，增长13.2%，消化库存316万平方米；金融机构不良贷款率为2.3%；为企业降成本30.8亿元；21项补短板重点工程加快推进，解决103万农村人口安全饮用水问题，完成207个中心村电网改造升级，新建4G移动通信基站4894座，新增光纤入户41.3万户，累计完成天然气管网建设1064.5公里。成立粤财普惠金融融资担保公司，小微企业融资难问题进一步缓解。

“放管服”改革持续深化。高新区（清城区）成立粤东西北首个行政审批局。加快相对集中行政许可权试点，推进“一门式一网式”政务模式改革，建成四级联动政务服务大厅1306个，完善市级行政审批事项标准670项，实现“一口受理，一窗通办”。建成网上中介超市管理平台，清理规范市级行政审批中介服务事项26项。动态调整部门权责清单。基本完成食盐监管体制和承担行政职能事业单位改革。清理规范事业单位公共服务事项清单，公布34家事业单位575项公共服务事项。成为全国十个“一带一路”最佳投资营商环境城市之一。全市新登记各类市场主体3.79万户，增长12.3%，其中企业增长25.9%。

农村综合改革深入推进。成立村民理事会16812个，成立经济合作社19602个，累计整合耕地面积154.2万亩，整合涉农资金41.6亿元，建成镇级农村产权流转管理服务平台85个、信用合作部57家，村级金融服务站100%覆盖，累计发放“两权”抵押贷款2.25亿元。扎实推进农村土地确权颁证，完成实测承包地面积403万亩，实测率105.9%，发证率95%。大力发展农村电商，建成电商村级服务站350个，“清远鸡”在天猫、京东年货节获评“最受欢迎年货”。培育壮大新型农业经营主体，新增农民合作社152家，家庭农场1934家。新增“三品一标”40个。清新创建为“国家级出口食品农产品质量安全示范区”。国家柑橘种苗繁育基地项目落户佛冈。

一年来，我们着力美丽清远建设，突出抓好环境整治和创文创卫，城乡面貌大为改善。

环境治理扎实有效。在全省率先建设“智慧环保”应用平台，在线监控企业128家。抓好中央环保督察问题整改，关停取缔整改企业168家。推进“创模”攻坚。全力推进空气环境“五大领域”治理，淘汰黄标车7333辆，完成陶瓷生产线“煤改气”32条，完成VOCs综合整治企业13家。全面推行河长制，设立四级河长1716名，清理禁养区内畜禽养殖场374个。清新与旧城污水处理厂二期基本完工，4条黑臭水体达到“不黑不臭”，完成龙塘河截污一期工程4.1公里，11个县级以上集中饮用水源水质100%达标。清理龙塘、石角遗留固废堆渣1.4万吨。

城市环境明显改善。创建成为国家卫生城市。以创文创卫为抓手，强化环境卫生、交通秩序、农贸市场和社区服务等突出问题综合整治，完成6大类3000多个整治建设项目，整治老旧小区3015个、内街背巷525条，解决一批群众关心关注的热点问题，市区面貌焕然一新；推广践行清远文明十二条，发动“礼让斑马线”行动，市民行为更加文明，文明卫生正成为构建清远更美好生活的坚实基础。起草我市第二部地方性法规《清远市城市市容和环境卫生管理条例》。中心城区扩容提质稳步推进，伦洲大桥、人民东路、铁塔路等建成通车，北江四桥主墩双主塔顺利封顶，清远大道、凤翔大道、城西大道等城市主干道升级改造完成，三大安置区建成交付使用，“四馆”前期工作基本完成，建成智能停车位3000个、充电桩54条。各县（市）城市建设和文明创建工作取得明显成效。

美丽乡村建设蓬勃开展。制定“美丽乡村2025”行动计划，实施五大创建工程，投入2.4亿元，建成美丽乡村802条，整治20户以上自然村8427条。成为全国美丽乡村建设示范区。3个镇（村）被评为第五届全国文明镇（村）。英德九龙镇河头村入选“中国美丽乡村百佳范例”。佛冈、连南入选省级新农村连片示范建设工程项目单位。全市共有省级宜居城镇19个、宜居村庄42个、宜居社区54个。完成碳汇造林4.2万亩，新建森林公园14个、湿地公园2个，建成乡村绿化美化示范村133条。乡村旅游加快发展。完成26.8万亩高标准农田建设、236.1公里中小河流治理、313公里县道“烂路”改造、400公里农村窄路拓宽。新增通客运班车行政村55个。

一年来，我们着力保障改善民生，突出抓好民生实事，社会事业全面发展。

民生实事全面完成。全市民生投入242.1亿元，占一般公共预算支出的79.2%。城镇、农村居民人均可支配收入分别增长8.9%和10%。改造农村危房7200户。棚户区改造住房新开工580套，基本建成4559套。培训创业创新人员1.18万人。完成100座危桥、200公里农村公路硬底化改造。境内普通公路全部取消收费。完成20个城中村污水管网改造建设。实施财政农业种养保险补贴项目17个。为95间乡镇卫生院、4间社区卫生服务中心配备中药处方库管理应用仪。全力攻坚精准扶贫，投入10.26亿元，启动村帮扶项目2221个，户帮扶项目47.3万个，5.73万贫困人口实现脱贫。

公共服务稳步提升。出台我市第一部政府规章《清远市教育设施规划建设管理规定》。3所学校入选首届全国文明校园。成为全省唯一全国未成年人思想道德建设工作先进市。清城、佛冈、连南成功“创先”，改扩建义务教育学校23所、新增学位5625个，实现义务教育学校标准化全覆盖，广清结对帮扶学校197所。大力推进卫生强市建设，公立医院全部取消药品（中药饮片除外）加成，家庭医生签约服务覆盖率40.3%，重点人群63.5%，13家医疗机构与广州建立结对帮扶关系。积极申办省第十六届运动会。成功举办清远马拉松、市第六届运动会、乡村戏曲节等群众文体活动。

社会保障更加健全。稳步提高城乡低保、特困人员供养、医疗救助等补助标准。加快社会养老服务体系建设。认真做好托养人员安置工作。进一步完善大病补充医疗保险制度。新增公共租赁住房446套，新增发放城镇住房保障家庭租赁补贴438户。举办各类就业专场招聘会324场次，新增城镇就业4.78万人，城镇登记失业率为2.37%。积极开展社保扩面征缴，参保总人次790.5万，超额完成目标任务。

一年来，我们着力政府自身建设，突出抓好依法行政，社会大局和谐稳定。

法治建设得到加强。认真落实市人大及其常委会各项决定，主动接受市政协民主监督，办理人大代表议案建议133件、政协提案289件。落实行政首长出庭应诉制度，行政机关负责人出庭应诉35次。严格执行市政府重大行政决策程序规定。完成新一届政府法律顾问换届选聘。大力推动政务公开，通过网络和报刊媒体主动公开各类政府信息12万余条。

作风建设深入推进。认真学习贯彻习近平新时代中国特色社会主义思想、党的十九大和习近平总书记对广东工作的重要批示精神，严格落实中央八项规定，狠抓“四风”整治，推动“两学一做”学习教育常态化、制度化。持续开展“正风”行动和“公述民评”活动，机关作风明显改善。认真贯彻全面从严治党新要求，强化党风廉政建设，行政监察和审计监督不断强化，政府系统惩治和预防腐败体系进一步健全。

社会大局保持稳定。深化平安清远建设，抓好特别防护期社会维稳工作，开展大接访活动，妥善解决一批历史遗留问题。劳动纠纷案件下降38%。严厉打击各类违法犯罪，深化全民禁毒斗争，积极推进“扫黄打非”，社会治安综合治理得到加强。安全生产形势总体稳定。食品药品、粮食安全保障能力有效提升。对外交流再结硕果，与新西兰达尼丁市正式建立友城关系，与巴拿马科隆市签署两国首个友城备忘录，首次获邀参加“中新市长论坛”。统计、民族、宗教、侨务、港澳、人防、国防动员、方志、档案、气象、水文、地震、慈善、妇女儿童、残疾人等工作取得新成绩。

各位代表，过去一年政府工作取得的成绩，是省委、省政府和市委正确领导的结果，是全市人民和社会各界凝心聚力团结奋斗的结果，是广州、佛山等兄弟城市大力帮扶支持的结果。我代表市人民政府，向全市人民，向驻清部队和各级驻清单位，向所有关心支持参与清远建设发展的各界人士致以崇高的敬意和衷心的感谢！同时，我们也清醒认识到，我市发展仍然面临不少困难和问题：一是城乡区域发展不平衡、不协调问题依然突出，城市发展较快，但农村整体发展水平相对滞后，脱贫攻坚任务艰巨；北部四县（市）人均GDP、全面建成小康社会指数与南部地区存在较大差距，南部各县（市、区）之间发展也不平衡。二是经济总量不大，产业质量不高，重大产业项目缺乏，工业投资不足，先进制造业和高新技术产业总量偏小，新动能培育还需加强，转型发展任重道远。三是财力不强，税源不足，财政收支矛盾突出，全市一般公共预算收入增幅低于支出增速10.2个百分点，人均财力仅为全省平均水平的60%左右。四是生态环保压力较大，环保基础设施不完善，环境监管能力有待加强，空气质量改善不明显，大气污染防治形势依然严峻。五是城市管理、社会治理能力有待提升，系统化、精细化、智能化管理水平不高，城市综合执法体制机制不完善，功能设施配套不足，停车难、交通拥堵问题日趋突出，市民文明健康素养还有待提升。六是基本公共服务水平总体不高，教育、医疗等优质资源不足、配置不均衡，群众性大型文体场馆建设滞后，与群众对美好生活的需要和全面建成小康社会的标准存在不少差距，改善民生任务艰巨。七是政府服务效能仍需提升，个别部门、少数公务员执行力不强，缺乏主动作为、敢于担当、攻坚克难的精神。对此，我们将高度重视，采取更有力的措施加以解决。

以习近平新时代中国特色社会主义思想

统领政府一切工作

习近平新时代中国特色社会主义思想是我们党我们国家必须长期坚持的指导思想，是全党全国人民为实现中华民族伟大复兴而奋斗的行动指南。当前清远正处在加快振兴发展、全面建成小康社会的关键时期，我们要以习近平新时代中国特色社会主义思想为统领，准确把握当前社会主要矛盾转化的阶段性特征，科学谋划未来一个时期的政府工作，把学习贯彻习近平新时代中国特色社会主义思想转化为推动清远振兴发展的具体举措，让习近平新时代中国特色社会主义思想在清远落地生根、开花结果。

——把握区域协调发展战略新要求，大力实施“南融北康”计划。发挥各地资源禀赋优势，大力发展县域经济，着力增强区域发展协同性、拓展发展空间、挖掘发展潜力。推动广清两市形成更加紧密的合作关系，推进广清一体化高质量发展，主动融入大广州城市群。积极参与粤港澳大湾区建设和“一带一路”建设。加快完善北部地区基础设施，大力探索发展特色农林、休闲旅游、清洁能源等绿色生态产业，推动优势资源向优势产业转变。支持民族地区加快发展。推进以人为核心的新型城镇化，加快农业转移人口市民化。

——把握经济高质量发展新要求，坚定不移推动产业转型升级。坚持发展第一要务，坚持质量第一、效益优先，坚定转型发展定力，加快构建实体经济、科技创新、现代金融、人力资源协同发展的现代产业体系。深化供给侧结构性改革。积极对接广深港科技创新走廊，提升清远国家高新区发展水平，加快打造粤北区域科技创新中心。谋划推进区域性大数据中心建设。实施质量强市和品牌带动战略，加快淘汰落后产能，提升水泥、陶瓷、再生金属等传统产业技术水平。推进全域旅游发展，打造国际化旅游城市。大力发展实体经济，将先进制造业打造成为我市的主导产业，推动产业集聚发展。加快省级职教基地建设，加强创新型、产业人才队伍建设。深化“放管服”改革，优化发展环境。

——把握乡村振兴战略新要求，持续深化农村综合改革。坚持把解决好“三农”问题作为工作重中之重，按照产业兴旺、生态宜居、乡风文明、治理有效、生活富裕的总要求，以深化农村综合改革为抓手，建立健全城乡融合发展体制机制，加快推进农业农村现代化。坚持质量兴农、绿色兴农，推进农业供给侧结构性改革，加快补齐“三农”发展短板，大力发展优质特色农产品，推动农业品牌规模效益发展。拓展农业产业形态，大力发展食品加工、农业电商、观光休闲农业、乡村旅游，促进一二三产业融合发展。推进美丽乡村建设，努力在全省率先实现美丽乡村建设全覆盖。

——把握建设美丽中国新要求，扎实推进生态文明建设。牢固树立绿水青山就是金山银山、蓝天白云就是人民最大幸福的理念，以生态优先和绿色发展为引领，在高水平保护中实现高质量发展。统筹推进生态经济发展、生态环境保护、绿色宜居城乡环境建设、绿色生活方式引导，把生态优势打造成为清远的核心竞争力。推进主体功能区建设，优化产业空间布局。加快粤北生态特别保护区规划建设。严守生态保护红线，全面加强环境保护和治理，落实“河长制”，强化大气、水、土壤污染治理。发展绿色金融。加大造林绿化力度，稳步提升森林覆盖率，让绿色成为城乡人居环境的底色，让生态文明成为群众自觉的追求。

——把握主要矛盾转化新要求，不断提升市民获得感、幸福感和安全感。准确把握当前我市各项工作面临的新形势、新问题、新机遇，坚持以人民为中心的发展理念，把人民对美好生活的向往作为我们的奋斗目标，着力解决发展不平衡不充分问题。加快补齐民生领域短板，推进教育、医疗卫生、文化体育等领域基本公共服务均等化建设，确保在幼有所育、学有所教、劳有所得、病有所医、老有所养、住有所居、弱有所扶上不断取得新进展。深入推进脱贫攻坚，确保如期高质量完成精准脱贫任务。加强和创新社会治理，建设法治清远、平安清远。

2018年是全面贯彻落实习近平新时代中国特色社会主义思想和党的十九大精神的开局之年，是决胜全面建成小康社会，实施“十三五”规划承上启下的关键一年，也是清远建市30周年的重要时期。从外部看，世界经济进入上升周期；习近平新时代中国特色社会主义思想为我国发展注入了新的强大动力，经济保持回暖向好态势；省推动区域协调发展、珠三角和粤东西北产业共建持续加力，粤港澳大湾区建设加快推进。从市内看，落后产能逐步淘汰，产业结构日趋优化；农村综合改革成效显现，农业经济稳步增长；清远长隆、广清产业园、省级职教基地、高速公路等一批重大项目投资建设进入高峰期，清远的发展迎来了新的战略机遇期。我们必须坚定信心，把握机遇，应对挑战，真抓实干，夯实全面建成小康社会基础。今年政府工作的总体要求是：坚持以习近平新时代中国特色社会主义思想为统领，深入贯彻落实党的十九大、中央经济工作会议和农村工作会议精神，认真贯彻省委十二届二次、三次全会精神，坚持稳中求进工作总基调，坚持新发展理念，聚焦高质量发展，围绕“1112”发展布局，深入实施创新驱动、乡村振兴、区域协调发展战略，扎实推进主体功能区规划、广清一体化“十三五”规划，坚持全面深化改革，坚决打好防范化解重大风险、精准脱贫、污染防治三大攻坚战，为如期全面建成小康社会奠定更加坚实的基础。

今年经济社会发展的主要预期目标是：生产总值增长6%左右；固定资产投资增长10%；一般公共预算收入增长7%；社会消费品零售总额增长10%；城乡居民收入增长略高于经济增长；城镇登记失业率控制在3.5%以内，居民消费价格涨幅控制在3%以内；社会保障、资源环境保护、节能减排降碳及安全生产完成省定目标。这些指标的设定，主要考虑我市产业结构调整压力比较大，要为调整结构、淘汰落后产能留出空间，把工作重心放到高质量发展上，推动我市经济社会发展真正从量的扩张转向质的提高。

2018年工作安排

按照经济社会发展预期目标和省、市重点工作部署，今年重点抓好以下十个方面工作：

（一）抓好供给侧结构性改革。保持转型发展战略定力，全面完成省定淘汰落后产能任务，防止地条钢等落后产能“死灰复燃”。促进房地产市场平稳健康发展，完善租购并举住房制度，推进“建鑫”住房租赁平台发展；加大住房保障力度，加快城区廉租保障房建设，完成1346户城镇住房保障家庭租赁补贴发放。积极防范和化解金融风险，加强政府债务管理，严厉打击非法集资行为。大力降低企业成本，研究推出新的降成本举措，再为企业减负30.6亿元。加快补短板工作，力争农村地区电压合格率98.8%以上，农村自来水普及率90%以上，光纤入户率75%以上，移动宽带普及率70%以上，城乡及交通干线4G网络全覆盖。积极参与对外经贸交流活动，鼓励企业参与“一带一路”建设，加快创建“国家级出口产品质量安全示范市”，建成清远跨境电商分拣中心。推进全域旅游示范区创建，编制全域旅游发展总体规划，推动一批景区创建4A级，建设一批自驾游旅居车营地，力争在旅游产业融合发展、旅游公共服务、基础配套建设、旅游宣传营销等方面取得新进展。着力培育万达广场、时代广场、美林广场等片区城市商圈，优化提升城市广场片区商圈，完善美林湖、省级职教基地、清远长隆等区域商业配套，推进城区2-3条特色商业街规划建设。引导大型流通企业和商业综合体开展促销活动。优化电商发展环境，建设一批电商物流集配中心，在金创电商产业园建立电商人才培育基地。加快广百南部物流园项目建设。

（二）深化与珠三角产业共建。进一步优化产业共建机制，调整南部区县帮扶关系，完成“两德”合作区的移交工作。推进广清产业共建前端孵化器建设，建立清远广州招商中心、清远企业广州公共办事处。针对重点区域、重点产业，强化招商引资力度，开展面向高新技术企业、上市公司的专场招商推介会，力争年内引进不少于160个产业项目。完成省定梯度转移珠三角项目和投资目标任务。围绕三年内南部四县（市、区）至少帮扶引进一个投资超20亿元、两个投资超10亿元项目的目标，推动一批龙头企业落户清远。继续推动园区提质增效发展，力争全年园区完成工业项目固定资产投资125亿元，实现规上工业增加值156亿元。加快推进广清产业园清城片区扩园工作，推进广清产业新城国家级产城融合示范区建设，力争年内新动工企业20家，试（投）产企业15家；加快佛冈、清新片区基础设施建设。做好广清产业园清城片区、连州产业集聚地扩区申报工作。推动“两德”园和英红园融合发展。深化广清交通互联互通，力争广清地铁等一批重大交通对接项目纳入广州市交通总体规划，推动广清大道南延线、太石路接花都平步大道、王子山互通立交工程等项目开工建设。探索承接广州铁路货运、专业市场和农产品深加工等城市功能疏解。继续深化金融、教育、医疗、旅游、农业等方面帮扶对接。

（三）深入实施创新驱动发展。制定“创新十条”实施细则。加大力度培育、引进高新技术企业，力争年内高新技术企业达210家以上。加强研发机构建设，力争组建新型研发机构1家以上，市级以上工程技术研究中心突破180家，规上工业企业研发机构覆盖率达20%以上，推动企业孵化器提升发展。大力引进培养科技创新人才，发挥华南863人才驿站、天安智谷人才大市场等平台作用，引导校企合作，强化与中大、华工等科研院校的全面合作，力争引进、培育3个以上创新创业科研团队。组织好第十届全国北斗高峰论坛等活动，争取承办第七届中国创新创业大赛地方赛事。力争专利申请量、专利授权量、发明专利授权量分别增长20%、15%和10%以上。全力扶持企业上市，力争1-2家企业IPO，8家以上企业上“新三板”，100家以上企业在区域性股权交易中心挂牌。大力发展实体经济，落实好省支持实体经济发展10项政策，加大企业帮扶力度，加强技改和增资扩产项目要素保障，力争推动100家以上规上企业开展技术改造、24个以上项目开展增资扩产，完成工业技改投资63亿元，新增25家规上工业企业。探索普惠性科技金融试点，推动银企合作。引导成立清远民营企业投资股份有限公司。推进中小微企业服务中心县（市、区）全覆盖。

（四）加大重点项目建设力度。安排省、市重点项目204项，年度计划投资328.9亿元。建成开放清远改革纪念馆。制定重大旅游项目扶持办法，加快推进清远长隆项目建设，力争完成投资17亿元；加快清晖南路延伸段、平安路、森林大道等道路建设，启动环城东路建设。推动神华国华动工建设。加快省级职教基地建设，确保5所省属高职院校全部动工建设，力争1-2所首期秋季建成，积极引进财经金融类本科院校。推动佛清从、汕湛、汕昆等高速公路项目加快建设，“高接高”建成通车，力争完成高速公路建设投资113亿元。确保北江四桥建成通车。力促广清城轨一期全面建成、北延线开工建设。加快北江航道扩能升级工程建设，力争完成清远、飞来峡水利枢纽二、三线船闸主体工程。加快推进磁浮旅游专线、英德通用机场建设。争取广清永高铁纳入国家相关规划。启动潖江蓄滞洪区建设。推动国省道公路服务区建设，完成全市普通国省道生命安全防护工程，加强道路交通安全黑点和隐患整治。加强土地利用管理，完成水田垦造5000亩，推进城乡建设用地增减挂钩指标市域内合理配置试点，加大闲置用地处置力度，积极盘活低效用地，启动3-5个有一定规模的“三旧”改造项目。

（五）加快北部地区绿色发展。推动广连高速、二广与清连高速连接线加快建设，加快连州通用机场、韶贺高速、韶柳铁路、二广高速连山至贺州支线等前期工作，加快国道323线连山、连南县城段、省道261线连山上帅至连南大麦山段、省道260线阳山文塔大桥至水口段等改建工程建设，力争国道323线阳山乳源交界至岭背段、国道234线连州茅结岭到三村段等动工改造，基本完成德建水库建设，推进龙船厂船闸重建。发挥民族地区政策优势，强化“三连一阳”共同招商,积极发展总部经济；推动民族工业园加快标准厂房及配套设施建设，引进落地一批项目。支持连州建滔工业园建设。深化落实与碧桂园集团开展精准扶贫全面合作，支持碧桂园在每个县（市、区）建设一个项目和一个星级旅游酒店。按照省的部署，积极稳妥推进粤北生态特别保护区规划建设，科学合理划定保护区范围。推进主体功能区、国家重点生态功能区建设。支持生态旅游业发展，办好连州国际摄影年展、连南盘王节、连山戏水节等节庆活动，着力提升民族风情、山水生态等旅游品牌影响力。支持因地制宜发展绿色农业，大力发展食品、农产品深加工，推进供港澳农产品基地建设，支持连南创建“国家级食品农产品质量安全示范区”、连山创建“国家有机农产品认证示范县”、连州创建“省农产品加工试点县”、阳山创建“省农业综合标准化示范县”。加快风电、光伏发电等绿色能源项目建设，推进一批小水电扩容增效改造。支持北部地区对接广州公共服务帮扶。支持连南、连山加快县城民族特色化改造。

（六）全面推进城市提质发展。以申办第十六届省运会为契机，大力推动城市建设。启动奥林匹克体育中心、“四馆一中心”、环城西路、松苏岭公园、南岸公园等一批项目规划建设，加快湖城大道、清晖路等在建项目建设，推进职教一路、森林大道地下综合管廊建设，启动智慧新城建设，力争启动西部一级公路建设。办好建市30周年纪念活动，总结好改革发展经验和成果。启动新一轮创文攻坚，推进全域创文、城乡一体化创文，完善创文长效常态机制，引导市民更广泛参与创文活动，养成更加文明的行为，确保创建全国文明城市年度取得更加优异成绩；启动新一届创建全国双拥模范城；进一步巩固创卫成果，做好国家园林城市复检工作。推进数字城管平台建设和升级，完善城市综合执法体制，推动提升城市管理水平。完成江南水厂一期建设，加快市区供水管网和滨江备用水源项目建设。逐步改善旧城地下管网。推动市区三个高速公路出入口优化规划建设。优化交通信号灯设置、路牌指引。加大公交投放力度，抓好市区汽车充电桩试点建设。进一步改善停车条件。完成220千伏岭塘变电站、110千伏广清城轨龙塘牵引站等输变电工程建设。推进“多规合一”，完成城市总体规划，完善城区控制性详细规划。推动各县（市、区）绿道建设。支持英德“一江两岸”、连州城西城北新区、佛冈龙凤新区、阳山城东新区建设，启动清新中以科技小镇建设，加快银盏温泉、龙颈足球、黄花文旅等特色小镇建设。

（七）大力推动乡村振兴发展。深化农村综合改革，加快推进农村耕地整治，基本完成农村承包土地确权颁证工作；继续推进涉农资金整合；推广农事服务超市模式，整合建设农村社会化综合服务平台150个；完善农村产权流转管理服务平台，促进土地有序健康流转；抓好村集体经济发展试点。实施“3个三工程”，培养发展新型经营主体，力争新培育农民合作社100家、家庭农场1000家、农业龙头企业8家以上。加大农业特色品牌培育力度，推动“清远鸡”“英德红茶”“连州菜心”“东陂腊味”等农产品做大做强，力争新增“三品一标”25个。积极拓宽农民与市场对接渠道。抓好“菜篮子工程”。办好2018年广东省茶产业大会。大力发展“互联网+农业”，推进农业与旅游、教育、文化等产业融合发展。建成启用广清农业众创空间一期。积极稳妥推进农村集体产权制度改革、供销合作社综合改革。引导农村信用合作部规范运作，稳步推进农村“两权”抵押贷款试点，积极推广“政银保”，组建农业投资公司，全面完成各县（市、区）农信联社改制农商行工作。开发水果种植、林业火灾保险，扩大农业保险覆盖面。实施“美丽乡村2025”行动计划，完成80%以上自然村环境综合整治，40%以上非贫困村创建成为美丽乡村。基本完成英德、连州、阳山省级新农村连片示范项目建设。推进“田园综合体”建设。出台促进乡村旅游发展意见，抓好12个省定重点扶贫乡村旅游开发，积极创建省级农业公园，举办清远首届乡村旅游节，着力培育一批乡村旅游品牌。按照“四好农村路”标准，完成550公里窄路拓宽改造、95.5公里乡道改造，力争打造一个“四好农村路”示范县。完成6.9亿元农网改造升级投资、184.8公里中小河流治理。加快推进高标准农田建设。

（八）强化生态环境治理。以抓铁有痕的决心打好污染防治攻坚战。推动“创模”攻坚，力争上半年各项指标全部或基本达标。推进七星岗、滨江迳口、连州龙潭寺、广州花都（清新）产业园等中央环保督察问题整改。加强大气污染防治，强化工业企业、道路扬尘、机动车尾气、秸秆焚烧、餐饮油烟等污染防治，继续推进陶瓷企业“煤改气”，加快推进市区大气环境监测站点优化。全面落实河长制，设立河长制公示牌，完善工作机制。加快工业集聚区集中污水处理设施建设、现有污水处理设施提标改造、禁养区畜禽养殖业清理等工作，推动港口和船舶污染物接收转运及处理设施建设。加快整县推进污水处理设施建设，确保如期完成省定任务。深化市区4条黑臭水体治理。推进4个国控点水质在线监测系统建设。加快龙塘、石角二、三期电子废弃物污染治理。严厉打击稀土矿违法开采。加快智慧环保二、三期建设，强化重点排污企业在线监测。全面推进大气、水、土壤等环境信息公开。完成市区生活垃圾应急填埋场建设，推进生活垃圾源头减量试点，启动清远市再生资源处理中心项目。加快森林碳汇、生态景观林带、森林进城围城、乡村绿化美化等林业生态工程建设，开展生态红线划定工作。

（九）提高保障和改善民生水平。深入推进精准扶贫、精准脱贫，深化脱贫攻坚政策举措，突出抓好产业扶贫、就业扶贫等帮扶工程，建立健全贫困户稳定增收脱贫长效机制。推进“厕所革命”。加大力度扶持创业就业,实现全市城镇新增就业4万人。继续推行大病关爱救助制度，推进社会养老服务、儿童福利体系建设。开展农村留守儿童保护关爱行动。推进社保扩面征缴，确保全年征缴率100%。稳步推进机关事业单位养老保险制度改革。大力推进教育“创先”，力争“创先”实现县（市、区）全覆盖。扩大公益普惠性学前教育覆盖面，加快解决义务教育“大班额”问题，启动普通高中优质化、多样化、特色化建设试点，促进民办教育规范特色发展。抓好卫生强市创建，推进健康清远建设，深化医药卫生体制改革，进一步完善分级诊疗制度，推动县级医院综合服务能力、镇村医疗卫生服务能力、医疗联合体等建设项目落地实施，推进东华镇中心卫生院升级建设，提升妇幼保健计生服务能力，加强基层全科医生队伍建设，稳步提高基层医务人员待遇。实施全民健身计划，承办好省第六届少数民族传统体育运动会，举办好清远马拉松、足球联赛、乡村戏曲节等群众文体活动。强化“黑名单”管理，提升食品药品安全监管水平。深化平安清远、法治清远建设，推动基层综合服务中心全覆盖。严格落实安全生产责任。全面推进“扫黄打非”进基层。抓好打假、打传、打私、禁毒工作。

（十）不断加强政府自身建设。以习近平新时代中国特色社会主义思想为统领，深入学习贯彻党的十九大、习近平总书记对广东工作重要批示及系列重要讲话精神，扎实开展“不忘初心、牢记使命”主题教育，坚定“四个意识”，坚决维护以习近平同志为核心的党中央的绝对权威和集中统一领导，在任何时候、任何情况下都坚决做到高举旗帜、坚定信念、拥戴核心。全面把握党的建设新要求，坚持从严管党治党，切实加强政府系统党组织建设。自觉接受人大监督、政协民主监督，严格执行各项决议决定，坚持重大事项向人大报告、政协通报制度，办好人大代表议案建议、政协委员提案。深化“放管服”改革，加快相对集中行政许可权试点，深化“一门式一网式”政务模式改革，进一步完善网上办事大厅服务功能，赋予县级政府和园区更多权限，完善市场准入负面清单制度，优化审批流程，继续清理规范行政审批中介服务，推进“数字政府”建设，为企业和群众提供更加便利高效的办事环境。推进市直从事生产经营活动事业单位改革、经济发达镇行政管理体制改革，完成国有林场体制改革。加强法治政府建设，提高行政机关负责人出庭应诉率，充分发挥政府法律顾问作用，全面推进政务公开。深化预算管理制度改革，打造更高层次“阳光财政”。推进审计监督全覆盖。加强作风建设和纪律建设，深入开展“正风”行动、“公述民评”等活动，继续整治“四风”问题，提升12345政府热线服务效能，构建“亲”“清”新型政商关系，营造清风正气、干事创业的良好氛围。加强统计、民族、宗教、外事、侨务、港澳、粮食、人防、国防动员、档案、方志、气象、水文、慈善、妇女儿童、残疾人等工作。

各位代表，在落实省十件民生实事的同时，市政府将全力办好今年的十件惠民实事：一是继续提升底线民生保障水平；二是提高边远山区教师生活补助标准；三是加大学前教育及中小学学位供给；四是加大力度开展农村危房改造；五是切实改善农村交通安全环境；六是不断提高基层医疗卫生保障水平；七是大力开展创业就业培训活动；八是为边远山区基层群众“送戏下乡”；九是进一步加强消防公共安全能力建设；十是为市民提供更多健康休闲去处。

各位代表，新时代，新作为、新发展。让我们高举习近平新时代中国特色社会主义思想伟大旗帜，更加紧密地团结在以习近平同志为核心的党中央周围，在省委、省政府和市委的正确领导下，不忘初心、凝心聚力、奋勇前行，为加快清远振兴发展、全面建成小康社会而努力奋斗！
